# Function matters: a review of terminological differences in applied and basic clicker training research

**DOI:** 10.7717/peerj.5621

**Published:** 2018-09-19

**Authors:** Nicole R. Dorey, David J. Cox

**Affiliations:** Department of Psychology, University of Florida, Gainesville, FL, United States of America

**Keywords:** Animal behavior, Behavior analysis, Animal training, Clicker training

## Abstract

In clicker training, animal trainers pair a small device (a “clicker”) with a reward when teaching or maintaining responding. Animal trainers often assume clicker training is a “science-based” way to train animals. But, the few studies that have compared clicker training to a control have not provided evidence that adding a clicker is beneficial to training. This may be because research on clicker training has studied only one of several potential functions of the clicker stimulus that have been discussed by animal trainers. A systematic approach to researching the function of the clicker in clicker training would benefit from collaboration between applied and basic researchers. However, this will require that terminological differences between animal trainers and basic researchers are reconciled. This paper reviews the few studies that have compared clicker training to a control group and then discusses how trainers and basic researchers use the same terminology in functionally different ways—suggesting the empirical support for mechanisms underlying clicker training is less robust than previously assumed. These differences highlight many opportunities to answer basic and applied research questions relative to clicker training methods. Advancements in clicker training methods will benefit animal trainers who have been using clicker training for decades as well as applied practitioners who have extended clicker training to humans in educational and clinical settings.

## Introduction to Clicker Training

Animal training has been around for thousands of years. Currently animal training is conducted by humans with a variety of species (e.g., aplysia–Vorster & Born, 2018; zebras–Deane, 2017), in a variety of environments (e.g., the home–Ziv, 2017; zoos–Colahan & Breder, 2003), and uses a variety of methods (e.g., Ziv, 2017; Deldalle & Gaunet, 2013). One of these methods is called clicker training because it typically uses a small plastic device called a clicker. The clicker produces a short, sharp two-toned clicking sound when the small metal piece is depressed. A click is typically presented immediately after a target behavior and before an already-established reinforcer is delivered. In the current paper, the terms click and clicker will be used generically to refer to a stimulus occurring after a target behavior and before an already-established reinforcer—even though the stimulus may take many forms (e.g., whistle, buzz). If a specific research study used a stimulus other than a clicker this will be made clear.

The first publication for the lay audience advocating the use of a clicker was featured in *Scientific American* (Skinner, 1951)*.* Skinner argued that using unconditioned reinforcers (e.g., food, water) may not allow for immediate delivery of reinforcement which could impact training success. He proposed a sound should also be emitted by the trainer, as opposed to other types of stimuli (e.g., visual), because sound can be experienced from a variety of locations and distances. Additionally, Skinner suggested that training begin by presenting the auditory stimulus followed immediately by food, which would likely cause the sound to become a conditioned reinforcer through Pavlovian processes (Skinner, 1951). This is often referred to by animal trainers as “charging the clicker” (see Alexander, 2003).

Skinner later demonstrated the use of a clicker-type device in 1952 when a journalist for LOOK magazine challenged Skinner to demonstrate the effectiveness of this method with a pet dog. Skinner subsequently met the reporter in one of his student’s apartments. The reporter brought a Dalmatian with him and asked Skinner to train her to “run up the wall” (Roddy, 1952, p. 17). In 20 min Skinner had trained the dog to jump up the wall using the flash of the camera as the clicker-type device.

For decades after Skinner’s (1951) article, clicker training remained primarily within the professional animal training sphere. The popularity of using this technique can be attributed to several individuals. Notably, Keller and Marian Breland (and later Bob Bailey) with the start of their business Animal Behavior Enterprises in 1947 (Bailey & Bailey, 1996), and Karen Pryor who adopted the use of operant conditioning and clickers from a manual written by Ron Turner (Pryor, 1999).

The current clicker training approach is defined as “an animal training method based on behavioral psychology that relies on marking desirable behavior and rewarding it” (Pryor, 2006a para 1). In addition to the use of clickers, clicker training includes the use of shaping to establish behavior, the presentation of discriminative stimuli (or “cues”) to signal which behavior will be followed by reinforcement, and the use of reinforcement rather than punishment (Ferguson & Rosales-Ruiz, 2001; Pryor, 2009).

With the roots of clicker training set firmly in behavior analytic research, it is not surprising that terminology between the two areas is similar. But, many terms used in clicker training (e.g., marking, bridging, and conditioned reinforcement) have not been used in the same way by basic researchers in behavior analysis (see sections further on that define each of these terms). In this paper, we start by reviewing the research that has been done comparing clicker training to a control group in an animal training setting. We then review the technical definitions of the proposed clicker functions from basic behavioral research. This comparison highlights some discrepancies in how the terms are used by some animal trainers and non-animal trainer researchers. We then provide solutions to areas of disconnect so applied and basic behavioral researchers can empirically advance the effective use of clickers in animal training.

## Literature Survey Methodology

**Figure 1:**
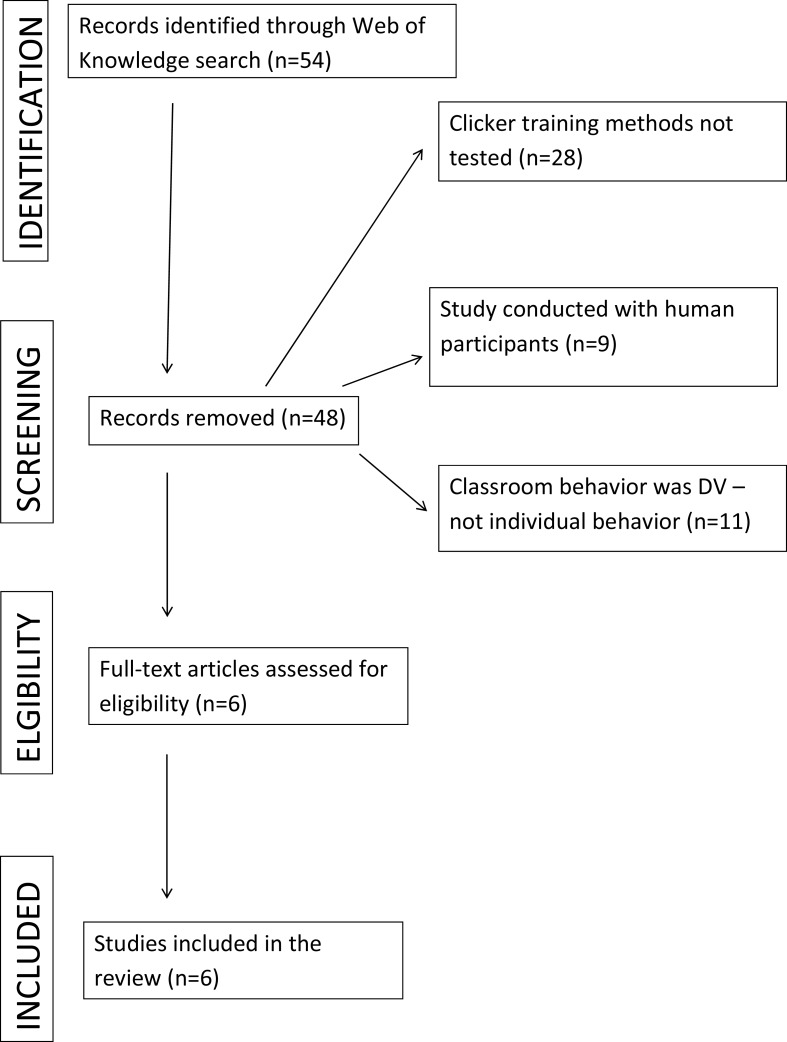
Flow diagram for the systematic review.

We started by reviewing what research has been done comparing clicker training to a control condition. We systematically searched the Web of Science database to identify peer-reviewed journal articles using the search term “clicker training”. Our search was performed in English for any research articles up to and including May 2017. The resulting articles were screened based on the primary dependent variable and population under study. We included studies that specifically measured the effectiveness of clickers in nonhuman animal training compared to a control group. Figure 1 shows a flow diagram for the systematic review following Prisma guidelines (Moher et al., 2009). Our search produced five peer reviewed studies.

## The Effectiveness of Using a Clicker during Animal Training

The first study to investigate the effectiveness of adding a clicker like device (buzzer) was conducted by McCall & Burgin (2002). These researchers examined if pairing a buzzer with food was more effective to train a new behavior than presenting food alone. The dependent variables in their study were the time required to train the new behavior and time until the new behavior ceased when extinguished. In their study, 48 horses were initially trained to press a lever. Next, all horses received 90 trials of training over three days. During this training period, half of the horses were placed in an experimental condition (buzzer paired with grain), and half in a control condition (grain only). After the third day of training, lever presses for both groups were extinguished (i.e., no food was delivered following a lever press), but the buzzer still followed lever presses for the horses in the experimental group. After the extinction test, all horses entered phase 3 where the buzzer was used to train a new behavior (flap press). The investigators found that the experimental group did not emit more responses or take longer to reach the extinction criterion in phases 1 or 2. The authors concluded the buzzer did not lead to any observable advantages in training efficiency or time until lever pressing stopped. However, the experimental group did emit significantly more flap presses in phase 3 than the control group.

Taking a similar approach, Williams and colleagues (2004) investigated whether adding a clicker reduced the number of trials required to train horses to touch a traffic cone with its nose. They randomly assigned 60 horses to one of six groups. For our purposes, the crucial comparisons involved three groups: horses that received only food for correct responding during training and nothing during extinction; horses that received food plus clicker during training and nothing during extinction; and, horses that received food plus clicker during training and a clicker during extinction. Williams et al. (2004) found that none of these groups acquired the target response more quickly or emitted more responses during extinction. These results supported those of McCall & Burgin (2002) suggesting that adding a clicker to the training procedures did not produce advantages when training horses to touch cones.

Smith & Davis (2008) extended the studies above to dogs using a three-phase procedure. During the training phase, dogs contacted reinforcement for successive approximations to touching a traffic cone with their nose. Dogs in the control group received food for successive approximations to cone touching whereas dogs in the clicker group received a click followed by food for successive approximations to cone touching. The second phase was a strengthening phase. In the strengthening phase, the dogs contacted the same reinforcement as the training phase for touching the cone but on a VR-2 schedule (i.e., every two responses were reinforced on average). The strengthening phase continued for 40 trials per day until a strengthening criterion was met. Once the strengthening criterion was met, the dog moved to the extinction phase. During the extinction phase, dogs in the control group received no programmed consequence for cone touches and dogs in the clicker group received only a click. Smith and Davis found no difference between dogs in the clicker group and those in the control group on the acquisition of the cone touching behavior. However, they did find that the clicker group took longer to meet the extinction criterion.

Thorn et al. (2006, Experiment 2) used a slightly different approach in their investigation. These researchers investigated whether presenting a click paired with food provided an advantage over presenting an alternative stimulus (verbal praise) paired with food. Thorn et al. taught two groups of shelter dogs to sit when approached by an unfamiliar person. One group of six dogs was trained for ten trials across two days where each instance of sitting was reinforced with the clicker followed by food. For the remaining six dogs, sitting was followed by the verbal phrase “good dog” followed by food. Dogs in both groups demonstrated similar latency reduction across trials on the first day. But, dogs in the clicker group showed increased latency to sit between the last trial of the first day and the first trial two days later. This suggests the added verbal stimulus was associated with a higher level of response maintenance than the added click stimulus. Nevertheless, both groups had learned to sit by the end of the second day of training.

In contrast to the above, one study found that a clicker facilitated learning in dwarf goats. Langbein et al. (2007) trained dwarf goats to complete a matching-to-sample task on a touch-screen. In the experimental group, goats that selected the correct shape were presented a tone followed by water. If they made an incorrect response, goats in the experimental group were presented only with a different tone. Goats in the control group were presented with water after correct responses, and did not contact any programmed consequence for incorrect responses. After several days of training, the performance of the two groups began to separate and goats in the experimental group required fewer trials to reach an accuracy criterion. However, the results of this study cannot be attributed solely to the click following correct responding as these goats also were presented a tone following incorrect responses.

Finally, Chiandetti et al. (2016) investigated whether presenting a click paired with food provided an advantage over presenting verbal praise paired with food, or food alone. They trained 51 dogs to push a handle up with their muzzle to open a bread box. After the dogs learned to perform that task, they trained the dogs to perform a slightly modified version of the behavior toward a different box to test for generalization. The investigators found no significant difference between trials to a learning criterion with the new box task when food was presented alone, with verbal praise, or with a clicker.

In sum, existing research suggests that adding a clicker to positive-reinforcement-based training does not present advantages above unconditioned reinforcers alone. Four out of the five studies found that presentation of a click with food did not lead to faster learning during training or better performance (McCall & Burgin, 2002; Williams et al., 2004; Smith & Davis, 2008; Thorn et al., 2006). The one study that did observe improved performance with a clicker also presented a tone following incorrect responses (Langbein et al., 2007). Providing a differential consequence for incorrect responses makes it difficult to determine if there were benefits of presenting the clicker following correct responding.

The above review of the literature should be read with caution for two reasons. First, the studies above examined clicker training of a simple target behavior followed closely in time and space (i.e., contiguously) by an established reinforcer. This likely differs from many training settings where the clicker is used precisely because an established reinforcer cannot be presented contiguously with the click and/or the response. Nevertheless, this further supports a central point of this article—more research on clicker training is needed. Second, most of the studies above examined clickers as potential conditioned reinforcers. But, animal trainers often suggest clickers serve stimulus functions other than as conditioned reinforcers. Thus, it is possible the research above failed to use the clicker consistent with its assumed function in animal training contexts.

## What is the Stimulus Function of the Click?

Pryor (1999) proposed three potential functions of the click: marking, bridging, and conditioned reinforcement. Some clicker trainers have compared these terms to the same findings from basic empirical research (Feng, Howell & Bennett, 2016; McCall & Burgin, 2002; Smith & Davis, 2008; Williams et al., 2004). However, the two groups differ in how they functionally use these terms which makes direct comparison difficult. This section discusses how these terms are used in basic behavior research and how they are functionally different from those used in clicker training.

### Bridging stimulus

Within basic research, a “bridging stimulus” has historically been used in conditioning procedures where a delay exists between a conditioned stimulus (CS) and an unconditioned stimulus (US). Here, researchers have studied if adding an additional stimulus between the CS and US will improve learning (e.g., rate of conditioned responding—CR; trials to criterion). The additional stimulus can be presented in two ways. In trace conditioning, the additional stimulus is presented for a portion of the delay between the CS and US. In delay conditioning, the additional stimulus is presented for the entire delay between the CS and US. The idea is that the additional stimulus promotes an association between the CS and US by “bridging” the temporal gap.

Trainers often describe the function of a clicker as a bridging stimulus. However, using a clicker as a “bridging stimulus” in training contexts differs from how a “bridging stimulus” is used in basic research. Basic researchers primarily examine bridging stimulus-stimulus relations (i.e., CS-US), and consistently find delay conditioning is more effective than trace conditioning for visual and auditory stimuli (e.g., Balsam, 1984; Rescorla, 1982; Williams, 1991). In contrast, trainers primarily examine bridging response–stimulus relations (i.e., target behavior-unconditioned reinforcer; Pryor, 1999); and, often use a short quick signal stating that longer signals, such as using the word “good”, cause slower acquisition of the target behavior (Jones, 2002; Pryor, 1999; Ryan & Mortensen, 2004).^1^ 1One might argue that, although the emphasis is on bridging response-reinforcer relationships, they really are targeting the relationship between the stimulus conditions immediately following a response and the reinforcer (i.e., a stimulus–stimulus relationship)—making bridging and marking similar. Even if true, a response is different than a response product (Palmer et al., 2004). Thus, the primary thesis of the manuscript remains supported—more research on clicker training is needed to establish what is being learned and the function of the clicker.Thus, stimulus presentations, responses being measured, and methods for improving learning that are associated with the term “bridging stimulus” are different across the two contexts.

### Marking stimulus

The marking hypothesis contends that presentation of a salient stimulus following a response will facilitate learning (Lieberman, Mcintosh & Thomas, 1979). In particular, the marking hypothesis suggests unexpected and novel events may cause an organism to attend to the response that immediately preceded it (Lieberman, Davidson & Thomas, 1985). Presenting an unexpected and novel stimulus immediately after a response (i.e., marking the response) will increase the likelihood the organism remembers the marked response when a programmed consequence occurs–instead of remembering and associating the delivered consequence with other responses emitted after the target response. Marking differs procedurally from bridging as the marking stimuli should be unexpected, novel, and uncorrelated with reinforcement (Lieberman, Mcintosh & Thomas, 1979). In addition, the marking hypothesis suggests marking stimuli should strengthen correct and incorrect responses similarly which differentiates marking from conditioned reinforcement (Hackenberg, 2009; Williams, 1991; Williams, 1994).

Previous research has supported the marking hypothesis. A robust observation from basic research is that inserting a delay between a response and the programmed consequence leads to slower response acquisition (e.g., Lieberman, Mcintosh & Thomas, 1979; Rachlin & Green, 1972). However, marking correct and incorrect behavior has been shown to improve learning when reinforcement is delayed compared to trials where marking does not occur (e.g., Lieberman, Mcintosh & Thomas, 1979; Lieberman, Davidson & Thomas, 1985; Thomas et al., 1983).

Animal trainers often use the term “marking” differently than basic researchers (Pryor, 2009). Basic research suggests marking stimuli should be unexpected, novel, and uncorrelated with reinforcement. However, the clicker in animal training is not unexpected, not novel, and is correlated with reinforcement. Many trainers “charge” the clicker before training by pairing it with food across multiple trials (Alexander, 2003; Smith & Davis, 2008) and strive for their animal to be “clicker-wise” or “clicker savvy” (Adams, 2003; Fisher, 2009; Pryor, 2009). The clicker also is only used to mark correct behavior, and animal trainers often discourage a “no reward marker” where the trainer clicks following an incorrect response (Pryor, 1999). Thus, although the terms are the same across both areas, the functional presentation of stimuli associated with these terms differs across the two contexts.

### Conditioned reinforcement

Conditioned reinforcers are initially neutral stimuli that become reinforcers as a result of having been paired with already-effective reinforcers (Catania, 2013). Unlike the terms “bridging” and “marking” stimuli, basic researchers and animal trainers seem to use “conditioned reinforcer” in similar ways. But, despite using the terms similarly, it is unclear that the clicker has been shown to serve as a conditioned reinforcer in animal training contexts.

Williams (1994) notes two general methods that researchers use to investigate how conditioned reinforcers affect behavior. One method involves presenting only the conditioned reinforcer contingent upon the occurrence of a new response. A second method is to compare resistance to extinction across two scenarios: (a) the unconditioned reinforcer is removed and a conditioned reinforcer is still presented following each response; and (b) the unconditioned reinforcer is removed and no stimulus change follows each response.

McCall & Burgin (2002) were the only researchers to test whether a clicker could be used to train a new behavior. Although they concluded they could train a new behavior using the conditioned reinforcer, there are some questions about whether the new behavior was trained through conditioned reinforcer properties of the clicker. For example, the “new” behavior trained was a flap press and the original behavior was a lever press. The flap press and lever press behaviors were topographically the same as both required the horse to push a manipulandum with their nose. The similarity in response topographies makes it hard to differentiate whether response acquisition was the result of stimulus generalization or the conditioned reinforcer properties of the clicker. A more convincing demonstration would have involved training a different, perhaps incompatible, response such as a rope pull in the presence of a significantly different discriminative stimulus.

Resistance to extinction was used in three of the experiments that investigated the function of the clicker during animal training (McCall & Burgin, 2002; Smith & Davis, 2008; Williams et al., 2004). Only Smith & Davis (2008) observed greater resistance to extinction in the clicker group compared to the group with no stimulus change following responding. As noted by Williams (1994), the overall change in environmental conditions during the extinction contingency for the clicker group is less than for the control group. This may influence the discriminability of the extinction condition in effect and could be one reason organisms might be more resistant to extinction in clicker versus non-clicker groups.

A further complication exists when analyzing the conditioned reinforcer properties of clickers. Some clicker trainers suggest that the trainer always pair food with the clicker (Alexander, 2003; Fisher, 2009; Martin & Friedman, 2011). This is often a primary component of the clicker trainer method. For example, Fisher (2009) writes, “Even if you goofed [accidently click the clicker following a behavior the trainer does not want to reward], pair a reward with your clicker”.

In sum, published research has not demonstrated that clickers are conditioned reinforcers. And, current training methods are unlikely to provide evidence that clickers are conditioned reinforcers. Thus, basic and applied research is sorely needed to determine the function of the clicker in animal training contexts.

## Future Directions to Test Proposed Clicker Functions

Basic research investigating the processes behind marking, bridging, and conditioned reinforcement suggest it is difficult that a single device could be all three. For example, basic research differentiates the functionally important stimulus relations between marking and conditioned reinforcement. Marking develops its stimulus function through proximity to correct and incorrect behavior. Conditioned reinforcement develops its stimulus function through the proximity of the stimulus to the unconditioned reinforcer (Hackenberg, 2009). In applied clicker training contexts, the click typically occurs in close proximity to both the behavior and the reinforcer (Pryor, 2009) making it difficult to separate marking from conditioned reinforcer.

When marking and conditioned reinforcer properties have been dissociated in basic research, proximity to the reinforcer appears to have the most influence on behavior. For example, Cronin (1980) used a two-key procedure with pigeons. Reinforcement was delayed by one minute after a correct response, and the trial ended without food one minute after an incorrect response. In the critical condition that separated marking from conditioned reinforcing functions of relevant stimuli, one light color occurred immediately after correct responses and a different light color occurred immediately before food. For incorrect responses the colors were reversed and food was not delivered. Cronin found the stimulus that had been presented immediately before food subsequently reinforced incorrect responses (i.e., an increase in the frequency of incorrect responses). This suggests the stimulus-reinforcer relation likely has a greater influence on response acquisition than the behavior-stimulus relation from a marking hypothesis. If it were the latter, no change in responding on the incorrect key would have occurred.

Williams (1991) also attempted to separate the effects of marking, bridging, and conditioned reinforcement experimentally in a two-choice, delayed-reinforcement procedure using rats. The correct lever was paired with a conditional cue. In the presence of white noise, presses to the left lever would produce food. In the presence of a light, presses to the right lever would produce food. In both the marking and bridging conditions, a tone was presented after responses to both levers but food only followed “correct” responses. In the marking condition stimuli were brief and immediately followed responses, while in the bridging condition tones were presented during the entire delay between the response and food. In the conditioned reinforcement condition, only correct responses were followed by the tone. Williams then compared trials-to-criterion from these three procedures to a condition where only food followed correct responding after a delay. Williams found that trials-to-criterion were the lowest in the conditioned reinforcer conditions whereas no difference was observed between the food only, marking, and bridging conditions. Thus, this research suggests the click might be most effective if used as a conditioned reinforcer.

The proximity of a conditioned reinforcer to a response also influences responding. Delaying any reinforcer reduces its efficacy (e.g., Nevin, 1974; Jarmolowicz & Lattal, 2011). Therefore, whether it is more important for a click to be presented immediately after the target behavior or immediately before food highlights an important direction for future research. Directly comparing these two variables is especially germane when there is likely to be a delay between the target response and presentation of an unconditioned reinforcer. That is, the shorter the delay between the behavior and the click the longer the delay between the click and unconditioned reinforcement. More basic research will be needed in this area to help clarify when best to present the click. To our knowledge no basic research studies to date have investigated the effects of notable delays to unconditioned reinforcement in a manner similar to how clickers are used in applied animal behavior training. Future researchers should replicate the procedure from Williams (1991) using clickers in animal training contexts.

As noted above, food is frequently delivered following every response in training contexts. Given the potential complexity involved in understanding the function of a clicker, another area of future research is whether the addition of a clicker is redundant. If a stimulus such as the appearance of food can be delivered easily, and is immediately experienced by the animal, then the addition of a click may offer no benefits to trainers. For example, Pryor (2009) described training a crab by feeding it pieces of food with forceps, and hoping that the movement of the forceps through the water—which immediately preceded the food—would also become a marking stimulus. It is unclear that adding a clicker is beneficial in these types of situations. Future research should examine the contexts where adding a clicker is beneficial and the contexts where it is unnecessary.

## The Click as a Discriminative Stimulus

It may also be useful to conceptualize the click as a discriminative stimulus. The click follows the target behavior, but it *precedes* another behavior required to obtain food. Fjellanger (2003) noted that charging the click should result in the dog running to get food immediately. In other words, the click should function as a discriminative stimulus indicating that food is now available following behavior necessary to locate and consume it. To our knowledge, the discriminative stimulus properties of the clicker has not been examined.

In studies that investigate clicker training to date, the food seeking behavior following a click has varied. For example, food seeking behavior has involved moving the head to take food from a bowl (Williams et al., 2004) or turning around and moving away from the location where the target behavior is emitted to pick food off the floor (Smith & Davis, 2008). Despite the inclusion of varying consumption responses, research has yet to focus on how different consumption behaviors influence clicker training and what stimuli affect consumption behaviors. It is also notable that most basic studies typically require minimal effort to retrieve the food once earned. This contrasts with applied contexts where the organism often engages in much greater effort to obtain and consume food once earned.

Charging the clicker may serve a similar role to magazine training in basic research. Magazine training involves teaching the organism that the stimulus complex associated with the food hopper is a discriminative stimulus for eating. Steinhauer, Davol & Lee (1976) demonstrated that the number of magazine trials pigeons experienced was negatively correlated with the number of autoshaping trials required to establish key pecking. Specifically, birds with no magazine training did not acquire key pecking after 100 autoshaping trials but pigeons with 25 magazine training trials acquired key pecking after a single trial. This supports the importance of the discriminative stimulus for food delivery in animal training contexts. Future applied researchers could extend basic research on magazine training to determine if charging the clicker similarly influences response acquisition.

One implication of a clicker functioning as a discriminative stimulus is that it may facilitate learning through a reduced overall delay to food. Basic researchers and animal trainers acknowledge that delay decreases reinforcer value (e.g., Rachlin & Green, 1972; Pryor, 2006b). For example, Pryor (2006b) states that food delayed by even five seconds after a click is presented may lead to “the animal … exhibiting new, untrained behaviors” (p. 25). These new behaviors likely are untargeted responses that occur during the delay between click and food, and are maintained through adventitious reinforcement. Using clickers as discriminative stimuli for food consumption behaviors may improve training outcomes by reducing the delay to unconditioned reinforcement. This would occur if the organism is taught to respond to the click by performing food acquisition behavior. This may reduce the overall time between click and food delivery and reduce the likelihood that untrained behaviors become adventitiously reinforced. Future studies could record delays between the target response and food in clicker and control groups to assess this possibility.

Viewing clickers as discriminative stimuli also fits with animal training situations as chained schedules. Described by Skinner (1938) and Ferster & Skinner (1957), each behavior in a chained schedule is followed by a stimulus change that may serve as a conditioned reinforcer for the behavior that preceded it, and as a discriminative stimulus for the next response in the chain. Chained responses occur until the last behavior is emitted and followed by an unconditioned reinforcer.

The standard clicker training situation could be viewed as a two-behavior chain. The target behavior produces the click. Then, food-acquisition behavior produces food. In an example potentially analogous to an animal training context, Kelleher & Fry (1962) demonstrated that response key light colors have both discriminative and reinforcing functions in a three-component, chained fixed-interval schedule. Future research in animal training contexts could assess a chained schedule hypothesis for clickers by using methodology similar to Kelleher & Fry (1962).

Other stimuli in the training environment also have the potential to serve combined discriminative stimulus/conditioned reinforcer roles. For example, Williams et al. (2004) initially trained horses to eat when food was placed in a bowl in their line of sight. The sight of the food and the trainer’s movements likely became a discriminative stimulus for food acquisition behaviors and may have acquired some conditioned reinforcing properties. When the click was added, learning may have been blocked by the already-acquired discriminative stimulus if the click did not precede the sight of food and trainer’s movements (e.g., Rescorla, 1988). These data highlight that understanding the processes involved in clicker training will require analysis of many different environmental stimuli within and preceding the training context. That is, how are various stimuli presented in relation to each other, in relation to behaviors and in relation to food. These factors likely will vary with the specific details of the training situation. This will add complexity to the analysis. But, it also highlights why developing a robust understanding of clicker training and the function of the clicker is important.

## Other Areas of Future research

A currently untouched area of future research in animal training contexts involves how best to thin unconditioned and conditioned reinforcers during acquisition and maintenance of target behaviors. Trainers often adjust the consequences they present following the target behavior as training progresses. In general, both the click and the food are presented on a continuous schedule while the target behavior is being established (see Alexander, 2003; Pryor, 1999). Once the behavior is established, dog trainers typically fade the click and present unconditioned reinforcement alone, and then end by presenting unconditioned reinforcement on a variable schedule. Animal trainers that work in a zoo environment use a different approach. They present the clicker alone following some responses, and present the clicker and food following some responses. This occurs when training a new behavior and when maintaining an established behavior (K Ramirez, pers. comm., 2010). The schedule of pairing clicks with food also becomes important. Just as with unconditioned reinforcers, intermittent schedules of conditioned reinforcers are likely to enhance the reinforcing value of the click the most (Williams, 1994). In addition, the effectiveness of the clicker as a reinforcer may be reduced if the unconditioned reinforcer is not presented intermittently over time (Rescorla, 1988). Future research should explore how differential patterns of thinning unconditioned reinforcers, conditioned reinforcers, and unconditioned-conditioned reinforcer pairings influences maintenance of learned behaviors in animal training contexts.

Another high impact future area of research is identifying the most efficient methods for establishing clickers as generalized conditioned reinforcers (Skinner, 1953). A generalized conditioned reinforcer is a conditioned reinforcer that has been paired with many different reinforcers such that its effectiveness does not depend on any state of deprivation (Catania, 2013). It is often recommended trainers pair the click with a variety of effective reinforcers so the effectiveness of clickers is unaffected by motivational states relevant to specific reinforcers (e.g., Alexander, 2003; Fjellanger, 2003). For example, a conditioned reinforcer paired only with food would likely be less effective if the animal had eaten recently. But a conditioned reinforcer paired with food, water, and access to conspecifics is likely to be effective under a greater range of conditions. Although Skinner proposed this concept over 50 years ago, there are still many unanswered questions about generalized conditioned reinforcers (Hackenberg, 2009). Clicker training may provide a cross-species context to address questions about generalized conditioned reinforcement with relevance to both basic and applied questions. An initial study could compare acquisition and maintenance of responding using clicks paired with food alone or using clicks paired with several unconditioned reinforcers.

A variant to standard clicker training that is advocated by some trainers is the use of a “no reward marker” (see Alexander, 2003). A “no reward marker” is a stimulus (such as the spoken word “wrong”) that is presented after incorrect responses while the click followed by food is presented after correct responses. Alexander noted this approach is endorsed by some trainers but did not recommend it in most circumstances because of a general objection to the use of (potentially) aversive stimuli in training. Langbein et al. (2007) presented a stimulus analogous to “no reward markers” when their dwarf goat subjects made incorrect responses. This led to better performance than a control group where only unconditioned reinforcement was presented following correct responses. However, performance was not compared to a condition in which an added stimulus (e.g., click) was presented after correct responses but not after incorrect responses. Additional research would help identify if there are conditions under which a “no reward marker” (i.e., positive punisher) would contribute to training outcomes.

## Conclusion

Clicker training is widely recommended and used, but rarely studied empirically. Some proponents of clicker training state the methods are grounded in peer reviewed research (Pryor, 2009). Recently Feng, Howell & Bennett (2016) concluded that, based on their brief review of the basic research, the clicker: “most likely function[s] in a reinforcing capacity, provided they are first paired with an unconditioned reinforcer to the extent required to imbue them with reinforcing capabilities, and provided this ‘charge’ is maintained so that the reinforcing properties are not extinguished. Less certain is whether they also have bridging and marking capabilities” (pg. 38).

The research Feng, Howell & Bennett (2016) used for comparison inadequately described the state of research relative to clicker training for several reasons. First, the research cited by Feng, Howell & Bennett (2016) came from highly controlled laboratory contexts with procedures that allow for explicit testing of the stimulus function. This contrasts with the highly complex and nuanced applied training contexts in which clickers are used. Second, the empirical research cited by Feng and colleagues disallows statements about clickers functionally serving as a marker or bridge stimulus. Finally, the basic research and applied research cited by Feng, Howell & Bennett (2016) led them to conclude that the clicker functions as a conditioned reinforcer even though the necessary research to demonstrate the conditioned reinforcing properties of the clicker has not been conducted.

Basic and applied research on clicker training is needed across several domains. Basic research is needed to establish the contexts in which clicker training as a package, and the click as a component, improves animal training outcomes and maintenance in a controlled environment. This research has not been done. Basic researchers could also investigate how the delay to food delivery that is common in training contexts impacts the function of the click. Investigations into the delay between the behavior and click as well as between the click and the food would be tremendously valuable for animal trainers. Basic and applied research on clicker training would improve current animal training methods and the overall welfare of these animals. However, for useful interchange to occur between researchers and clicker trainers, it is necessary both groups are aware of the differences in how terms for describing the clicker training process are used. We hope this paper is a first step toward greater collaboration and opens the door to the vast amount of research needed in this area.

## Additional Information and Declarations

### Data Availability

The following information was supplied regarding data availability: This is a literature review. The research in this article did not generate any data or code.
